# Research on the Frequency Response and Dynamic Range of the Quadrature Fiber Optic Fabry–Perot Cavity Microphone Based on the Differential Cross Multiplication Demodulation Algorithm

**DOI:** 10.3390/s21186152

**Published:** 2021-09-14

**Authors:** Baokai Ren, Jin Cheng, Longjiang Zhao, Zhenghou Zhu, Xiaoping Zou, Lei Qin, Yifei Wang

**Affiliations:** 1 Research Center for Sensor Technology, School of Applied Sciences, Mechanical Electrical Engineering School, Jianxiangqiao Campus, Beijing Information Science and Technology University, Beijing 100101, China; renbk2021@163.com (B.R.); xpzou2014@163.com (X.Z.); qinlei@bistu.edu.cn (L.Q.); yifeiwang2020@126.com (Y.W.); 2 College of Engineering, Qufu Normal University, Rizhao 276826, China; honghuzlj@163.com; 3 School of Materials Science & Engineering, Nanchang University, Nanchang 330031, China; zhuzhenghou@nuc.edu.cn; 4 Beijing Key Laboratory for Optoelectronic Measurement Technology, Beijing Information Science & Technology University, Beijing 100192, China; 5 Key Laboratory of Modern Measurement & Control Technology, Ministry of Education, Beijing Information Science & Technology University, Beijing 100192, China

**Keywords:** quadrature Fabry–Perot cavity, differential cross multiplication, dynamic range, frequency response, quadrature phase deviation

## Abstract

A quadrature fiber optic Fabry–Perot cavity microphone based on a differential cross multiplication algorithm consists of a pair of fibers and a membrane. It has many advantages such as high sensitivity, a simple structure, and resistance to electromagnetic interference. However, there are no systematic studies on its key performance, for example, its frequency response and dynamic range. In this paper, a comprehensive study of these two key parameters is carried out using simulation analysis and experimental verification. The upper limit of the frequency response range and the upper limit of the dynamic range influence each other, and they are both affected by the data sampling rate. At a certain data sampling rate, the higher the upper limit of the frequency response range is the lower the upper limit of the dynamic range. The quantitative relationship between them is revealed. In addition, these two key parameters also are affected by the quadrature phase deviation. The quadrature phase deviation should not exceed 0.25π under the condition that the demodulated signal intensity is not attenuated by more than 3 dB. Subsequently, a short-step quadrature Fabry–Perot cavity method is proposed, which can suppress the quadrature phase deviation of the quadrature fiber optic Fabry–Perot cavity microphone based on the differential cross multiplication algorithm.

## 1. Introduction

As a new type of microphone, the fiber optic microphone (FOM) has the advantages of high sensitivity, low propagation loss, and resistance to electromagnetic interference compared with the existing electric, piezoelectric, capacitive, and other traditional acoustic microphones [1,2,3,4]. Therefore, it have a wide range of applications, especially irreplaceable applications in acoustic source detection under a strong electromagnetic environment [5].

FOMs have been extensively investigated. Various FOMs have been developed, including intensity-type, wavelength-type, and interference-type [6,7,8]. In recent years, interference-type FOMs have become a research hotspot, especially FOMs based on Fabry–Perot (F–P) interferometers [9,10,11], which are of great interest due to their high sensitivity, simple structure, and easy fabrication. However, the dynamic range of the fiber optic F–P cavity microphone is largely limited by the intensity demodulation method based on a linear region of the interference spectrum [12,13,14]. To extend the dynamic range of the fiber optic F–P cavity microphone, a quadrature signal demodulation technique has been proposed, which consists of the following two main aspects [15,16,17,18]:

1. Quadrature signal acquisition: The methods to obtain two quadrature signals mainly include the phase generated carrier (PGC) method [19,20,21,22], multiwavelength method [23,24,25], construction of quadrature F–P cavity method [26,27,28,29], etc. The PGC method is susceptible to the effects of external factors such as drift of mixing signal amplitude and change of phase modulation amplitude. In addition, the traditional PGC method is not suitable for short cavity interferometers, as with most monochromatic sources it is not possible to provide sufficient modulation depth due to the structural characteristics of short cavity interferometers. The multiwavelength approach requires optical filters or multiple light sources of different wavelengths, which complicates the sensing system and prevents the detection of high frequency signals due to the limitations of the spectrometer scanning speed [28]. In contrast, the method to obtain the quadrature signals by constructing a quadrature F–P cavity structure is simple, easy, and inexpensive. The method also solves the issue of reduced sensitivity due to changes in the initial cavity length of the F–P cavity.

2. Demodulation of quadrature signals: For the quadrature signal demodulation techniques, demodulation algorithms commonly adopted the differential cross multiplication algorithm (DCM) [30,31] and the arctangent algorithm [32,33]. For the quadrature F–P cavity structure, the arctangent algorithm cannot eliminate the effect of the initial phase on the demodulated signal. In addition, the arctangent algorithm involves relatively complex arctangent nonlinear operations and suffers from phase entanglement. The DCM algorithm has relatively simple differential operations without the phase entanglement and a high demodulation efficiency. The characteristics of the quadrature signal acquisition scheme and the signal demodulation algorithm scheme are summarized in Table 1.

Quadrature fiber optic Fabry–Perot cavity microphones based on the DCM signal demodulation algorithm (Q–FFPM–DCM) have good development prospects due to the advantages of their excellent performance, simple structure, and low cost. However, there are quite limited systematic studies on their key performance. The current research is concerned with the stability of the quadrature fiber optic Fabry–Perot cavity microphone (Q–FFPM) solution and the optimisation of the diaphragm structure and materials, but there is no complete analysis of the corresponding frequency range and dynamic range [28,34,35]. In this paper, the frequency response and dynamic range of Q–FFPM–DCM are studied systematically using simulation and analysis methods. The factors influencing the frequency response range and dynamic range of the microphone are systematically analysed and the quantitative relationships between sampling rate, frequency response range, and dynamic range are obtained. Subsequently, the effect of the quadrature phase deviation on the demodulated signal is comprehensively investigated by a data simulation method, and it is concluded that the quadrature phase deviation cannot exceed 0.25π under the condition that the intensity of the demodulated signal is not attenuated by more than 3 dB. Finally, a method to suppress the quadrature phase deviation caused by laser wavelength drift is proposed, and the correctness of the simulation analysis is verified experimentally.

## 2. Fundamentals of the Q–FFPM–DCM

### 2.1. Theoretical Model of the Quadrature Fiber Optic F–P Cavity Microphone

**Figure 1:**
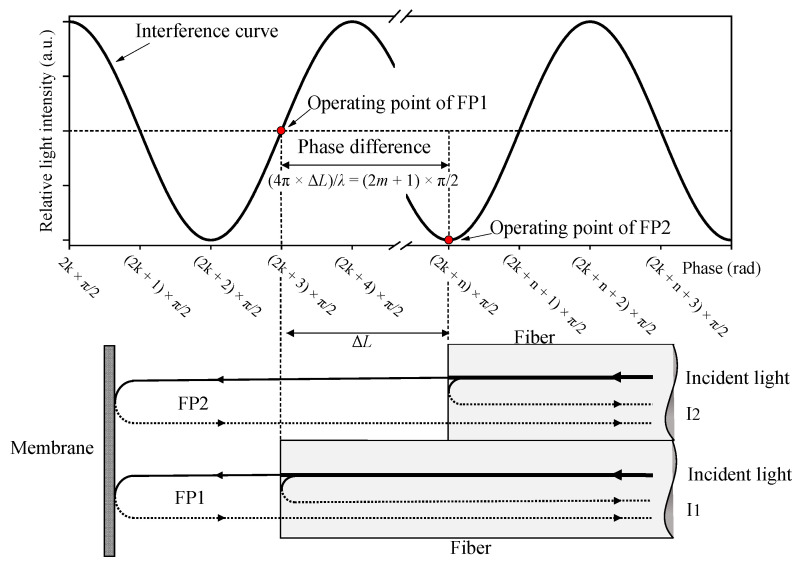
Schematic diagram of the quadrature fiber optic F–P cavity microphone.

The schematic diagram of the quadrature fiber optic F–P cavity microphone is shown in Figure 1, which consists of a fiber pair and a diaphragm. The height of step between the facets of fiber pairs is Δ*L*. In order to generate a quadrature signal from the two F–P cavities, Equation (1) should be satisfied.

(1) $$\frac{4\pi \times \Delta L}{\lambda }=2m+1\times \frac{\pi }{2}\;$$

where *m* is a nonnegative integer and λ is the wavelength of the laser.

### 2.2. DCM Signal Demodulation Algorithm

The light intensity of the interference spectrum of the two fiber optic cavities is $I_{1}\left(t\right)$ and $I_{2}\left(t\right)$. The photodiode can be used to convert the optical signal into an electrical signal, and then the capacitive filter is used to filter out the DC component of the signal, then the two output signals can be expressed as

(2) $$V_{1}\left(t\right)=A\cos\left[\Delta \varphi \left(t\right)+\varphi_{0}\right]\;$$

(3) $$V_{2}\left(t\right)=A\cos\left[\Delta \varphi \left(t\right)+\varphi_{0}+\frac{\pi }{2}\right]=A\sin\left[\Delta \varphi \left(t\right)+\varphi_{0}\right]\;$$

where *A* = $k\gamma RI_{0}$,  k is the photoelectric conversion factor; γ is the interference spectrum contrast; *R* is the resistance value of the photoelectric conversion circuit; $I_{0}$ = ($I_{max}$+$I_{min}$)/2, $I_{max}$ and $I_{min}$ are the maximum and minimum interference light intensity, respectively; Δφ(*t*) = $\varphi_{m}$ cos(*ωt*−$\psi_{0}$) is the phase change caused by a sound wave, which has round frequency *ω* and initial phase $\psi_{0}$; $\varphi_{0}$ is the initial phase of the interferometric spectrum.

The derivation of Equations (2) and (3), respectively, gives the following results,

(4) $$\frac{d\left(V_{1}\left(t\right)\right)}{dt}=-\frac{d\left(\Delta \varphi \left(t\right)\right)}{dt}\;A\sin\left[\Delta \varphi \left(t\right)+\varphi_{0}\right]$$

(5) $$\frac{d\left(V_{2}\left(t\right)\right)}{dt}=\frac{d\left(\Delta \varphi \left(t\right)\right)}{dt}\;A\cos\left[\Delta \varphi \left(t\right)+\varphi_{0}\right]$$

After cross multiplication and division, the result is shown as follows:

(6) $$V\left(t\right)=V_{2}\left(t\right)\frac{d\left(V_{1}\left(t\right)\right)}{dt}-V_{1}\left(t\right)\frac{d\left(V_{2}\left(t\right)\right)}{dt}\;$$

Then integrating Equation (6), the sound wave signal was obtained, as below:

(7) $$\Delta \varphi \left(t\right)=\frac{1}{A^{2}}\int^{\;}V\left(t\right)dt+B$$

where *B* is the integration constant.

## 3. Analysis of the Frequency Response and Dynamic Range

For microphones, the frequency response range and dynamic range are two key performance indicators. In this section, the effects of signal frequency, intensity, and data sampling rate on the signal demodulation of the Q–FFPM–DCM have been simulated. The relationship between the upper limit of the dynamic range, the upper limit of the frequency response range, and the data sampling rate was obtained through simulation. The parameters of the simulation are shown in Table 2. The relationship between the signal frequency, signal intensity, and data sampling rate was studied and analysed respectively using the control variable method as follows.

### 3.1. Simulation of the Effect of the Signal Intensity on Demodulated Signals

**Figure 2:**
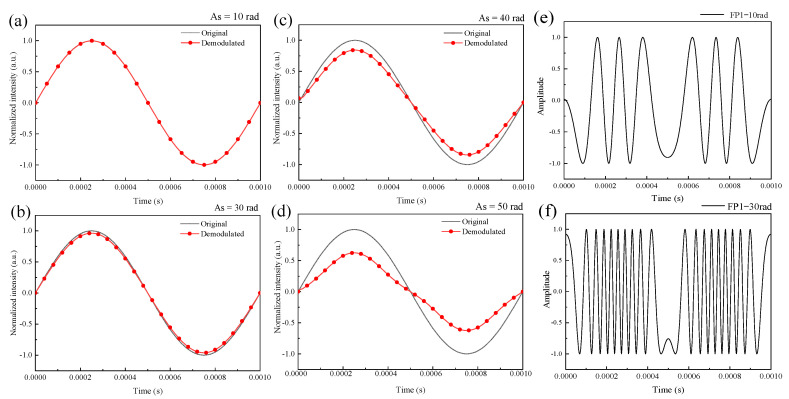
Simulation of demodulated signals with sound wave signal amplitudes of 10 rad (**a**), 30 rad (**b**), 40 rad (**c**), 50 rad (**d**) and the output signal of the FP1 cavity at signal amplitudes of 10 rad (**e**), 30 rad (**f**).

The frequency of the sound wave signal was set to 1 kHz and the data sampling rate was 100 kHz. The demodulated signals were simulated at 10 rad, 30 rad, 40 rad, and 50 rad, corresponding to Figure 2a–d respectively. From the simulation results, it can be seen that the normalized intensity of the demodulated signal decayed as the intensity of the sound wave signal increased. This phenomenon is related to the periodicity of the interference spectrum. The amplitude of the diaphragm vibration increases with the intensity of the sound signal increasing, correspondingly, the amplitude of the phase change increases. This results in heavy folding of the output signal from a single F–P cavity as shown in Figure 2e,f. At a finite sampling rate, the demodulated signal undergoes intensity attenuation as the individual F–P cavity output signal folds to the point where it does not satisfy the sampling theorem.

### 3.2. Simulation of the Effect of Signal Frequency on the Demodulated Signals

**Figure 3:**
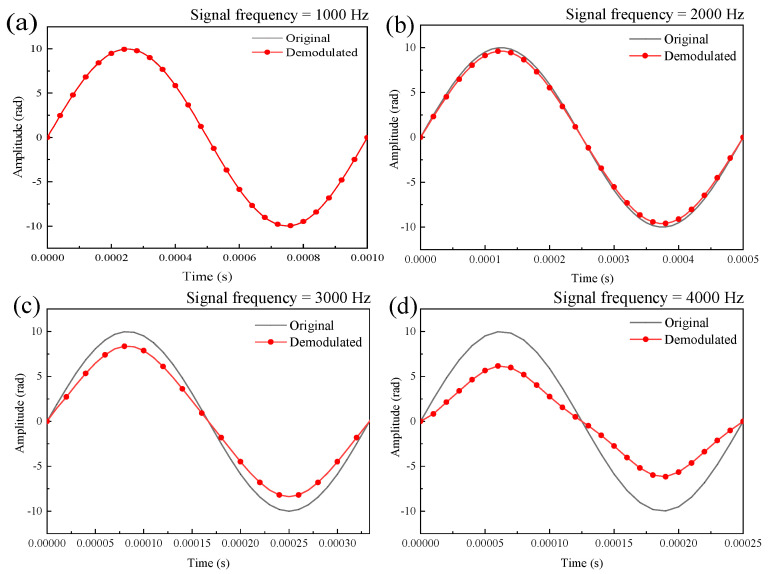
Simulation of demodulated signals at signal frequencies of 1000 Hz (**a**), 2000 Hz (**b**), 3000Hz (**c**), 4000 Hz (**d**).

The sound wave signal amplitude was set to 10 rad and the data sampling rate was 100 kHz. The simulated demodulated signals at frequencies of 1000 Hz, 2000 Hz, 3000 Hz, and 4000 Hz correspond to Figure 3a–d respectively. From the simulation results, it can be seen that the intensity of the demodulated signal was attenuated as the frequency of the sound wave signal increased. Similarly, as the frequency of the sound signal increased, the folding of the output signal from the single F–P cavity intensified and, with the limited sampling rate, also led to an attenuation of the intensity of the demodulated signal.

### 3.3. Simulation of the Effect of the Data Sampling Rate on the Demodulated Signals

**Figure 4:**
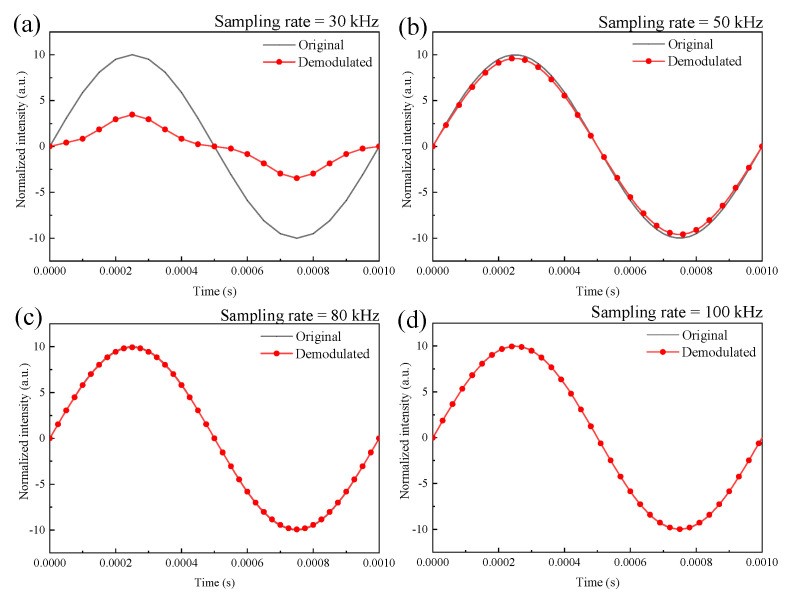
Simulation of the demodulated signals at sampling rates of 30 kHz (**a**), 50 kHz (**b**), 80 kHz (**c**), 100 kHz (**d**).

For data acquisition systems, demodulation resolutions of the Q–FFPM–DCM are largely determined by the data sampling rate. To investigate the effect of the data sampling rate on the Q–FFPM–DCM, signal demodulation simulation experiments were carried out at different data sampling frequencies. Setting the signal frequency at 1 kHz and the signal amplitude at 10 rad, the simulated demodulated signals at sampling rates of 30 kHz, 50 kHz, 80 kHz, and 100 kHz are shown in Figure 4a–d respectively. It can be seen that as the data sampling rate decreased, the demodulated signal also underwent intensity degradation. As can be seen from Figure 4a, when the data sampling rate was 30 kHz, the demodulated signal underwent severe intensity attenuation.

### 3.4. Relationship between the Upper Limit of the Dynamic Range of the Signal, the Upper Limit of the Frequency Response Range, and the Data Sampling Rate

The quadrature fiber optic F–P cavity microphone uses a nickel–metal diaphragm with a thickness of 3 μm and a diameter of 9 mm. The mechanical sensitivity of the diaphragm $S_{D}$, can be calculated as 19.81 nm/Pa based on the characteristics of the diaphragm [36]. The sound signal can then be expressed as

(8) $$\Delta \varphi \left(t\right)=A_{s}\sin\left(\omega_{s}t+\varphi_{s0}\right)=\frac{4\pi \times S_{D}\times P}{\lambda }\sin\left(\omega_{s}t+\varphi_{s0}\right)\;$$

where $A_{s}$ is the amplitude of the phase change in the interference spectrum caused by the sound wave signal (in rad); $\omega_{s}$ is the frequency of the sound wave signal (in rad/s); *λ* is the operating wavelength of the light source, which was set to 1550 nm; and *P* is the sound wave pressure of the sound wave signal (in Pa)

The data sampling rate ($f_{sam})$ was set to 10–200 kHz and the amplitude of the phase change of the interference spectrum caused by the sound wave signal ($A_{s})$ to 10–50 rad. Using the frequency at which the output signal attenuation reached 3 dB as the upper limit of the frequency response range, the simulation yielded the upper limit of the frequency response range $\left(f_{c}\right)$ of the demodulated signal; and the ratio (C) of the upper limit of the frequency response range to the signal sampling rate was calculated, as shown in Table 3.

Table 1 shows that under the condition that the demodulated signal intensity is not attenuated by more than 3 dB, the upper limit of the amplitude of the phase change of the interference spectrum caused by the sound signal, the upper limit of the frequency response range, and the system signal sampling rate are related by Equation (9).

(9) $$A_{s-max}=k_{1}\times \frac{f_{sam}}{f_{c}}\;$$

By substitution Equation (10) is obtained.

(10) $$P_{max}=k_{1}\times \frac{\lambda }{4\pi \times S_{D}}\times \frac{f_{sam}}{f_{c}}=k_{1}\times k_{2}\times \frac{f_{sam}}{f_{c}}\;$$

where $k_{1}$ = 0.258, the value of $k_{1}$ is taken as the signal intensity attenuation criterion. The value of $k_{2}$ depends on the light source wavelength and the mechanical sensitivity of the diaphragm; for this quadrature fiber optic F–P cavity microphone, $k_{2}$ = 6.226.

Different from the sensitivity mechanism of the capacitive electret microphone, the Q–FFPM–DCM works at the principle of light interference. Due to the periodicity of the interference spectrum, the output light intensity will change periodically when the sound intensity increases. This is obviously different from that of the sensor based on the monotonic variation law. For sensors based on the monotonic variation law, their output always increases with the intensity of the sound, however, it is effectively limited by the supply voltage. The periodicity of the interference spectrum is the physical basis of the Q–FFPM–DCM. Theoretically, the DCM can demodulate the acoustic signal with any sound pressure level. However, the output signal of the Q–FFPM will be folded continuously due to the periodicity of the interference spectrum when the sound pressure increases continuously. For data acquisition, this is equivalent to the frequency increasing. When the data are folded to not satisfy the sampling theorem, the DCM will not demodulate the original signal correctly due to the limitation of the data sampling rate. This is the physical essence of the influence between the dynamic range and the frequency response range. The upper limit of the dynamic range, the frequency response range, and the data sampling rate are restricted by Equation (10).

## 4. Effect of Quadrature Phase Deviation on Frequency Response and Dynamic Range

For quadrature fiber optic F–P cavity microphone, the demodulation of sound signals based on the differential cross multiplication algorithm is most accurate when the phase difference between the quadrature F–P cavity signals is an odd multiple of π/2. However, errors can occur in the wavelength drift of the light source, resulting in quadrature phase deviations of the quadrature F–P cavity signals. In the following, a description is given of the simulations of the demodulation of the signals in a nonexact quadrature state that were carried out to analyze the effect of the deviation of the quadrature phase on the demodulation of the signals.

### 4.1. Simulation of the Effect of the Quadrature Phase Deviation on the Frequency Response

**Figure 5:**
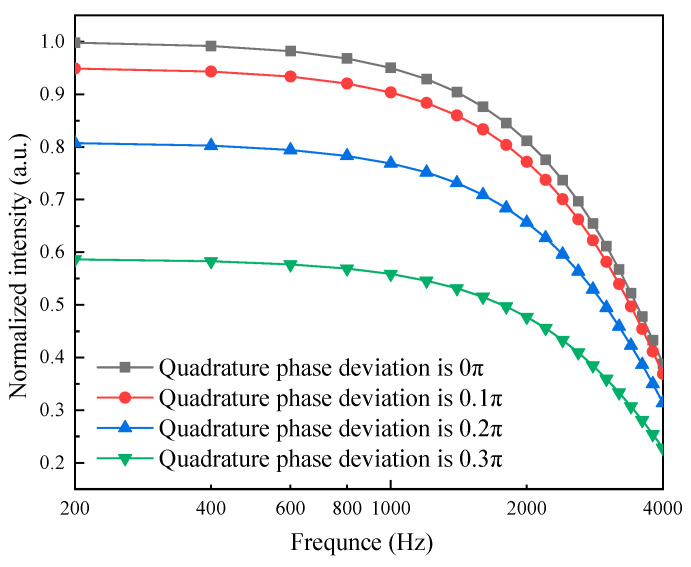
Frequency response curves for different quadrature phase deviations.

To investigate the effect of quadrature phase deviations on the frequency response of the Q–FFPM–DCM, simulations were carried out in the frequency range from 200 Hz to 4000 Hz by setting the quadrature phase deviation to 0π, 0.1π, 0.2π, and 0.3π, respectively, and the amplitude of the sound wave signal to 10 rad, and the simulation results are shown in Figure 5. It is obvious that when the quadrature phase deviation increased, the frequency response range of the quadrature fiber optic F–P cavity microphone decreased accordingly.

### 4.2. Simulation of the Effect of the Quadrature Phase Deviation on the Dynamic Range

**Figure 6:**
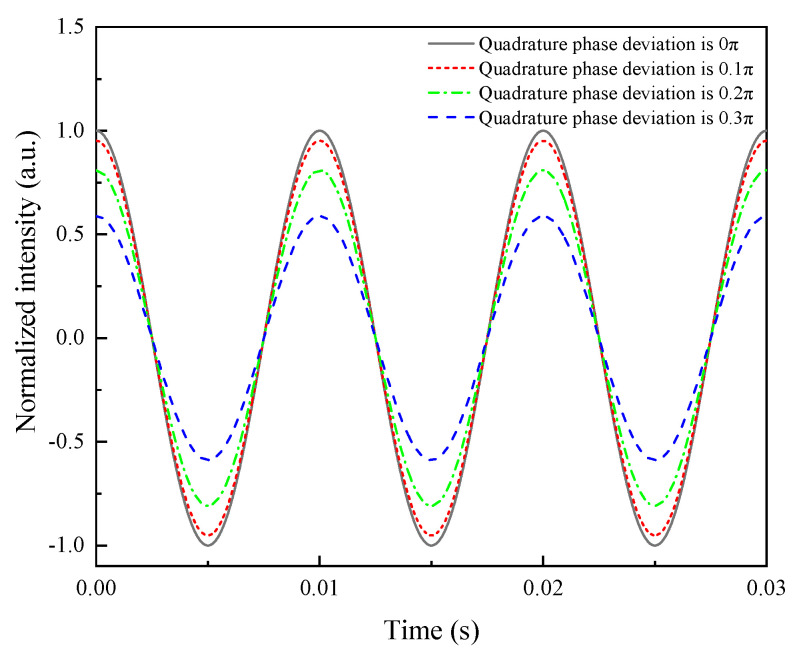
Demodulation effect of signals with different quadrature phase deviations.

**Figure 7:**
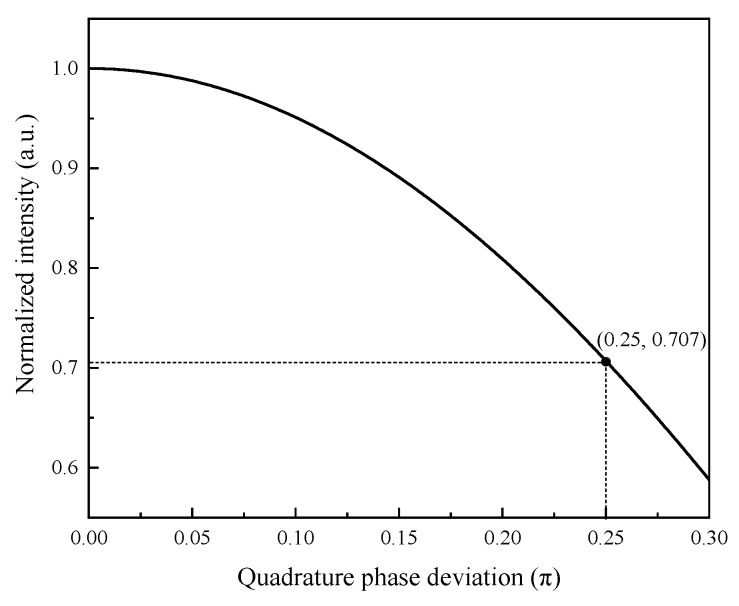
Demodulated signal intensity versus quadrature phase deviation curve.

The demodulation simulation results for a sound pressure of 100 Pa, a frequency of 100 Hz, and a sampling rate of 100 kHz with quadrature phase deviations of 0π, 0.1π, 0.2π, and 0.3π are shown in Figure 6. Clearly, the smaller the quadrature phase deviation, the more accurate the signal demodulation of the Q–FFPM–DCM. If the demodulated signal intensity was attenuated by no more than 3 dB as the condition for effective demodulation, the demodulated signal failed at the quadrature phase deviation of 0.3π. The range of the quadrature phase deviation was set to 0–0.3π, and the demodulated signal intensity curve was plotted as shown in Figure 7. The maximum quadrature phase deviation to make the demodulation effective was 0.25π. This indicates that the quadrature phase deviation of the quadrature F–P cavity signals should be less than 0.25π to achieve effective demodulation using the DCM algorithm in practical applications.

### 4.3. A Method to Suppress the Quadrature Phase Deviation

In practice, semiconductor lasers are used as monochromatic light sources for quadrature fiber F–P cavity microphones, whose wavelength varies with temperature, which is the main source of quadrature phase deviation. Laser light source wavelength drift experiments were carried out and showed that the laser wavelength drift measured over a temperature range of 0 to 40 °C reached 2.682 nm. In order to suppress the quadrature phase deviation caused by the laser wavelength drift of the laser, a method of short-step quadrature F–P cavity is proposed.

**Figure 8:**
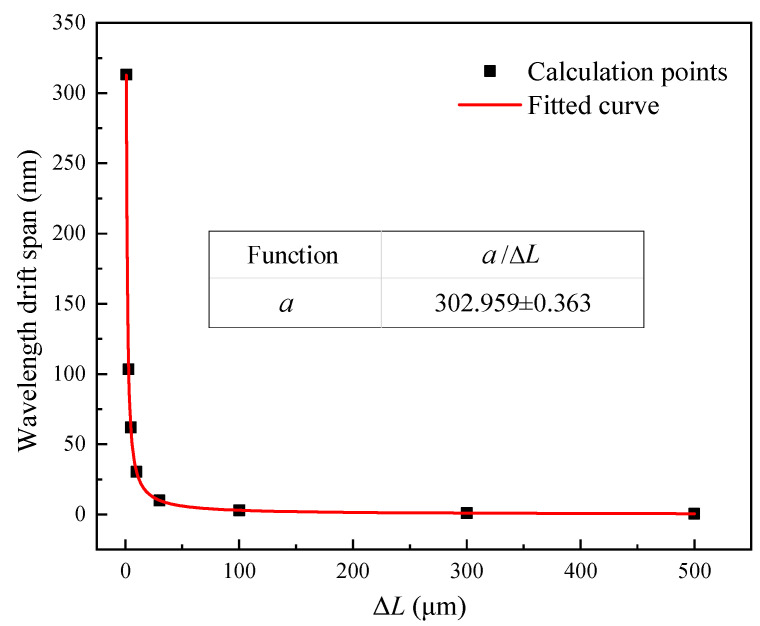
Fitted relationship between the step height and the permissible wavelength drift span.

According to the basic principle of the F–P interferometer, the phase difference between the two F–P cavity signals should be an odd multiple of π/2. The relationship between the wavelength of the light source (*λ*) and the step height (ΔL) of the quadrature F–P cavity can be obtained as in Equation (2). Under the condition that the quadrature phase deviation does not exceed 0.25π, the operating wavelengths of the light sources corresponding to different ΔL and their permissible drift ranges were calculated, as shown in Table 4. We know that the permissible wavelength drift span (*PWDS*) increases significantly as the step height decreases at the operating wavelength of 1550 nm. So the quadrature phase deviations can be depressed by reducing the step height of the quadrature. This method has the advantages of extending the operating temperature range and reducing system cost. The relationship between *PWDS* and ΔL is obtained by data fitting as shown in Figure 8.

## 5. Experiment

### 5.1. Q–FFPM–DCM Probe Construction and System Components

**Figure 9:**
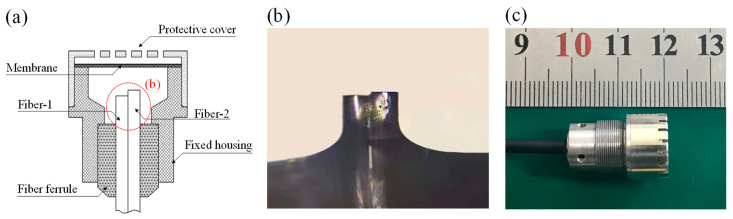
Schematic diagram of Q–FFPM probes (**a**). Microscopic photo of a short step fiber pair (**b**). The diameter of the fiber shown in Figure 9b is 125 μm. Digital photo of a Q–FFPM probe sample (**c**).

A Q–FFPM–DCM probe was fabricated, which consisted of just a single mode short fiber step pair, a fixed housing, a fiber ferrule, a membrane, and a protective cover, the structural composition of which is shown in Figure 9a. The membrane was made of nickel with a thickness of 3 μm and a diameter of 9 mm. A short step height (Δ*L*) of 4.82 μm is shown in Figure 9b. The view of the sensor probe appearance is shown in Figure 9c.

**Figure 10:**
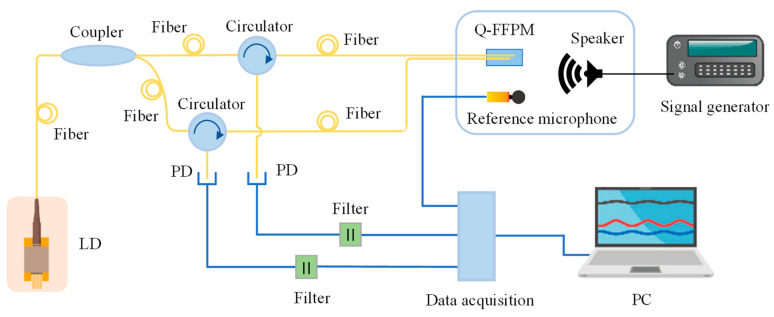
Q–FFPC–DCM experimental system composition diagram.

The sensor system was built as shown in Figure 10. The monochromatic light from the ZH-550D DFB laser diode (LD) was divided into two beams by the coupler into the quadrature F–P interference cavity, respectively, and the two returned interference signals carrying sound information then passed through the circulator to the photodiode (PD), which converted the light signal into an electrical signal. The two electrical signals passed through the capacitor filter, respectively, filtering out the DC component of the signal and entering the MIC-7700 data acquisition system. Finally, the acquired signal entered the computer, which ran the LabVIEW program to realise the demodulation of the sound signal. The GRAS 46AZ microphone was used as a reference and was arranged side-by-side with the Q–FFPM–DCM probe for simultaneous acquisition of the sound signal.

### 5.2. A Method for Adjusting the Q–FFPM–DCM into Quadrature State

**Figure 11:**
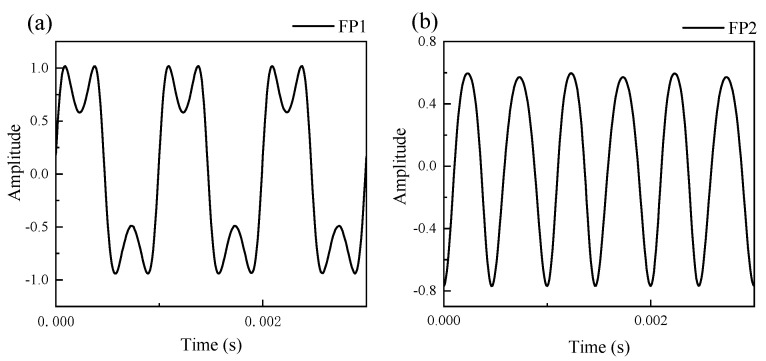
Determination of Q–FFPM–DCM quadrature state dual F–P cavity reference output. The output signal of FP1 (**a**) and FP2 (**b**) cavity in quadrature state.

The quadrature state of the Q–FFPM–DCM is its working basis. According to the principle of differential cross multiplication, the phase difference between the operating points of the two F–P cavities should be an odd multiple of π/2. Due to the periodic nature of the F–P cavity interference spectrum, at a suitable sound pressure, when the operating point is at an odd multiple of π/2 of the interference spectrum, the output signal will show the symmetrical concave output phenomenon shown in Figure 11a. When the operating point is at an even multiple of π/2 of the interferometric spectrum, the output signal will appear as a multiplicative output as shown in Figure 11b. At this time, the phase difference between the operating points of the two F–P cavities was exactly an odd multiple of π/2, i.e., the sensor was in quadrature. In the experiment, by changing the wavelength of the laser, the operating points of the two F–P cavities drifted at the same time under changing ambient temperature, and observing the output signal, if the waveform shown in Figure 11 appears, then the sensor was in the quadrature state at that wavelength.

## 6. Experimental Results and Discussion

### 6.1. Experimental Results at Different Signal Frequencies

**Figure 12:**
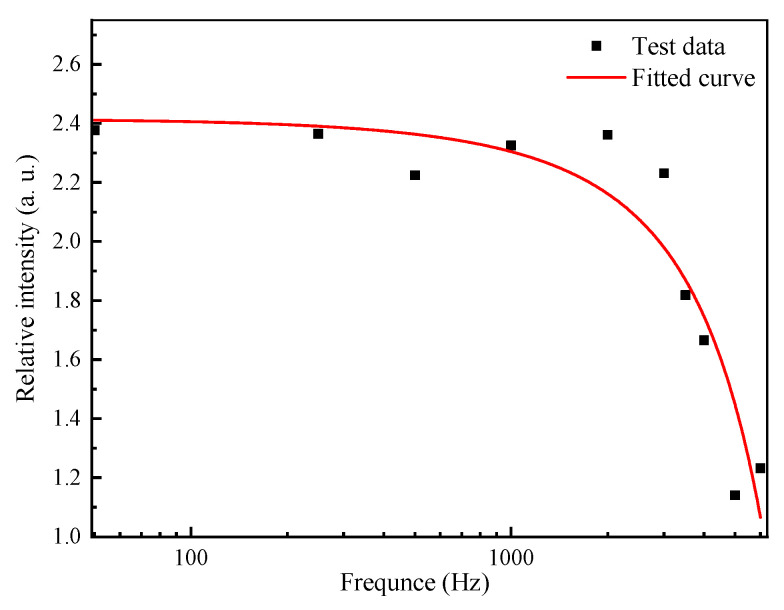
Plot of demodulated signal intensity versus sound wave signal frequency.

In order to verify the variation of the demodulated signal of quadrature fiber optic F–P cavity microphone under the action of sound waves of different frequencies, the demodulation results of the sensor were tested at 10 frequencies in the frequency range of 50 Hz–5 kHz in the experiments. Based on the 10 sets of measured data, the output signal intensity ratio of the quadrature fiber optic F–P cavity microphone and the reference capacitive microphone could be obtained under the action of different frequency sound wave signals. As can be seen in Figure 12, as the frequency of the sound wave signal increased, the intensity of the demodulated signal decayed.

### 6.2. Experimental Results at Different Signal Intensities

**Figure 13:**
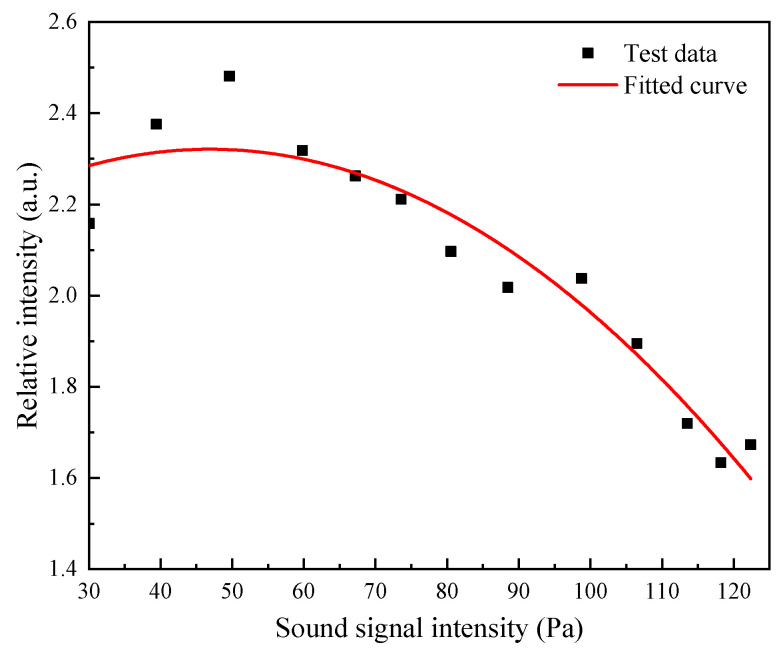
Plot of demodulated signal intensity versus sound wave signal intensity.

Setting the sound wave signal frequency to 1200 Hz, the relationship curve between the output signal intensity ratio of the quadrature fiber optic F–P cavity microphone and the reference capacitive microphone and the demodulated signal intensity was plotted at different sound wave signal intensities, as shown in Figure 13. From the experimental results, it can be seen that as the sound wave signal intensity increased, the relative intensity of the signal demodulated by the quadrature fiber optic F–P cavity microphone decayed.

### 6.3. Experimental Results at Different Data Sampling Rates

**Figure 14:**
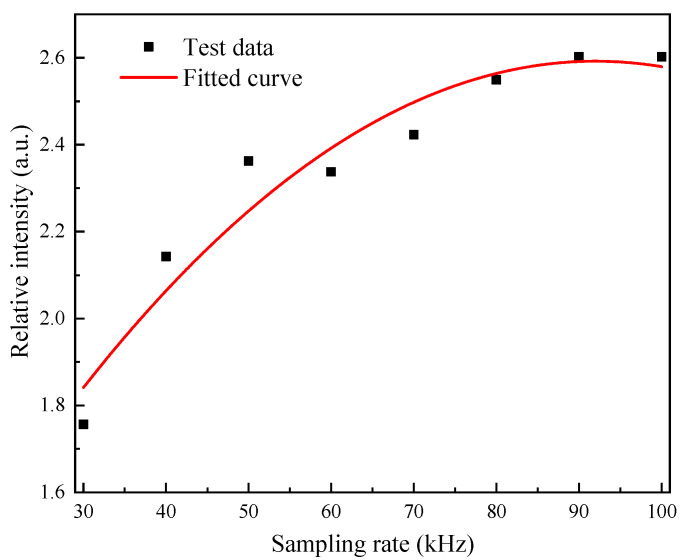
Plot of demodulated signal intensity versus data sampling rate.

Setting the sound wave signal frequency to 1 kHz and the sound intensity to 132 Pa, the output signal intensity ratio of the quadrature fiber optic F–P cavity microphone and the reference capacitive microphone was plotted against different data sampling rates as shown in Figure 14. It is clear that as the data sampling rate decreased, the demodulated signal intensity also decreased.

### 6.4. Results of the Suppression of the Quadrature Phase Deviation by Fiber Step Height

**Figure 15:**
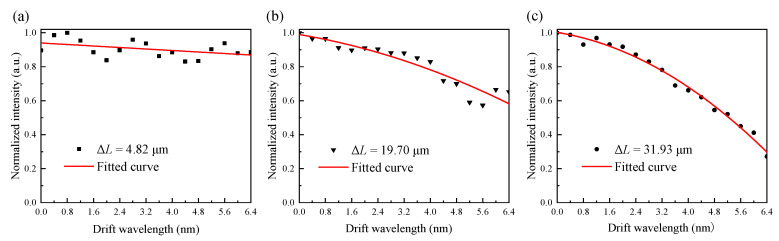
Q–FFPM–DCM demodulated signal intensity with wavelength drift for fiber step heights of 4.82 μm (**a**), 19.70 μm (**b**), 31.93 μm (**c**).

To investigate the suppression of the quadrature phase deviation caused by the laser wavelength drift, three Q–FFPM–DCM probes with fiber step heights of 4.82 μm, 19.70 μm, and 31.93 μm were fabricated and adjusted to the quadrature state before the experiment. The output wavelength of the tunable laser was adjusted in steps of 0.4 nm to take each of the three probes out of quadrature in order to increase the quadrature phase deviation. From the experimental results shown in Figure 15, the Q–FFPM–DCM had the highest suppression of the laser wavelength drift when the step height was 4.83 μm. The normalised intensity fluctuation shown in Figure 15a originates from the power fluctuation of the tunable laser. As shown in Figure 15b,c, the unilateral permissible wavelength drift range increased by approximately 1.3 nm as the fiber step height decreased from 31.93 μm to 19.7 μm. It is clear that the ability of Q–FFPM–DCM to suppress the quadrature phase deviation caused by the laser wavelength drift gradually increases as the fiber step height decreases.

### 6.5. Discussion

Experimental results have verified the results of the simulation analysis. There is a mutually constraining relationship between the dynamic range and the frequency response range of the Q–FFPM–DCM, both of which are limited by the sampling rate at the same time. The quantitative relationship between the three was derived and can be used as a reference for the design of the Q–FFPM–DCM system. In addition, the effect of the quadrature phase deviation on the Q–FFPM–DCM was systematically investigated. The qualitative relationship between the permissible wavelength drift range and fiber step height was given, which can be used as a reference for the design of Q–FFPM–DCM structure parameters. The validity of the short-step quadrature Fabry–Perot cavity method was verified by comparing the ability of different fiber step height Q–FFPM–DCMs to suppress the quadrature phase deviation caused by the laser wavelength drift. The directions of this paper are aspects that must be considered in the design and manufacture of the Q–FFPM–DCM and will have a positive effect on the realisation of a wide range of Q–FFPM–DCM applications.

## 7. Conclusions

This paper systematically discussed the frequency response and dynamic range of Q–FFPM–DCM and their influencing factors through simulations and experimental analysis. These two parameters are both influenced by the data sampling rate. The relationship between the upper limit of dynamic range and the upper limit of frequency response is negative under a certain data sampling rate. The quantitative relationship between them was obtained by simulation analysis and expressed as Equation (10). The three parameters are interlocked. The corresponding third parameter can be determined from knowing two of them. In addition, the effects of the quadrature phase deviation on the dynamic range and frequency response range were systematically studied. The quadrature phase deviation should not exceed 0.25π under the condition that the demodulated signal intensity is not attenuated by more than 3dB. A short-step quadrature Fabry–Perot cavity method is proposed to suppress the quadrature phase deviation caused by the wavelength drift of the laser source. A larger permissible wavelength drift range of the laser source can be obtained by reducing the step height. The results of the analysis show that the permissible wavelength drift span of the source increases from 2.997 nm to 62.024 nm when the step height is reduced from 100.16 μm to 4.84 μm.

## Figures and Tables

**Table 1:** Quadrature signal acquisition and demodulation scheme comparison.

System Features	Q–FFPM/DCM [26,27,28,29,30,31]	Q–FFPM/Arctan [32,33]	PGC/DCM [19,20,21,22,28]	PGC/Arctan [28,32,33]	Multiwavelength/DCM [23,24,25,28]
Number of light sources	1	1	1	1	≥2
Acquisition of quadrature signals	Simple	Simple	More complex	More complex	Complex
System costs	Low	Low	High	High	High
Algorithm complexity	Low	High	Low	High	Low
Whether a carrier is required	No	No	Yes	Yes	No

**Table 2:** Parameters of the simulation.

Symbol	Value	Description
*λ*	1550 nm	Wavelength
*L*_1_	375.18 μm	Initial length of short F–P cavity
*L*_2_	380 μm	Initial length of long F–P cavity
Δ*L*	4.82 μm	Fiber optic step height
k	0.95 A/W	Photoelectric conversion factor
γ	0.98	Contrast of interference spectrum
*R*	100 kΩ	Resistance value of the photoelectric conversion circuit
*I*_0_	10.7 μw	Central light intensity of the interference spectrum
*ψ*_0_	0	Initial phase of the sound signal
*n*	1	Refractive index of air

**Table 3:** Upper limits of the frequency response range of demodulated signals at different sound pressure and sampling rates.

$A_{s}\;(\mathrm{rad})$	$C\;(=f_{c}/f_{sam})$	$f_{sam}\;(\mathrm{Hz})$	10 k	20 k	50 k	79 k	200 k
10 (62.26 Pa)	0.0258	$f_{c}$ (Hz)	258	697	1292	2038	5160
20 (124.52 Pa)	0.0129	$f_{c}$ (Hz)	129	348	645	1019	2580
30 (186.78 Pa)	0.0086	$f_{c}$ (Hz)	86	232	430	679	1720
40 (249.04 Pa)	0.0064	$f_{c}$ (Hz)	64	174	322	509	1290
50 (311.30 Pa)	0.0051	$f_{c}$ (Hz)	51	139	258	407	1032

**Table 4:** Operating wavelength under different Δ*L*.

Δ*L* (μm)	Lower Limit of Wavelength Drift (nm)	Working Wavelength *λ* (nm)	Upper Limit of WaveLength Drift (nm)	Wavelength Drift Span (nm)
0.96	1409.091	1550	1722.222	313.131
4.84	1519.607	1550	1581.632	62.024
9.88	1534.951	1550	1565.346	30.395
30.03	1545.016	1550	1555.016	9.999
100.16	1548.502	1550	1551.500	2.997
500.06	1549.699	1550	1550.300	0.600

## Data Availability

The data used to support the findings of this study are available from the corresponding author upon request.
